# mFOLFIRINOX versus mFOLFOX 6 as adjuvant treatment for high-risk stage III colon cancer - the FROST trial: study protocol for a multicenter, randomized controlled, phase II trial

**DOI:** 10.1186/s12885-024-11939-x

**Published:** 2024-03-29

**Authors:** Kyung-Ha Lee, In Jun Yang, Gi Won Ha, Jaeim Lee, Youn Young Park, Suk Hwan Lee, Jong Min Lee, Jung Hoon Bae, Eun Jung Park, Hyungjin Kim, Keun Young Kim, Sanghyung An, Ik Yong Kim, Ji Yeon Kim

**Affiliations:** 1 Department of Colorectal Surgery, Chungnam National University Hospital & College of Medicine, Daejeon, Korea; 2 Biomedical Research Institute, Jeonbuk National University Hospital, Jeonju, Korea; 3 Department of Surgery, Uijeongbu St. Mary’s Hospital, College of Medicine, The Catholic University of Korea, Uijeongbu, Korea; 4 Department of Surgery, Kyung Hee University Hospital at Gangdong, Kyung Hee University College of Medicine, Seoul, Korea; 5 Department of Surgery, Yongin Severance Hospital, Yonsei University College of Medicine, Yongin, Korea; 6 Department of Surgery, Seoul St. Mary’s Hospital, College of Medicine, The Catholic University of Korea, Seoul, Korea; 7 Division of Colorectal Surgery, Department of Surgery, Gangnam Severance Hospital, Yonsei University College of Medicine, Seoul, Korea; 8 Department of Surgery, Eunpyeong St. Mary’s Hospital, College of Medicine, The Catholic University of Korea, Seoul, Korea; 9 Department of General Surgery, Wonkwang University Hospital & School of Medicine, Iksan, Korea; 10 Department of Surgery, Yonsei University, Wonju College of Medicine, Wonju, Korea

**Keywords:** High-risk stage III colon cancer, Adjuvant chemotherapy, mFOLFIRINOX, mFOLFOX 6, Prognosis, Efficacy, Toxicity

## Abstract

**Background:**

High-risk stage III colon cancer has a considerably poorer prognosis than stage II and low-risk stage III colon cancers. Nevertheless, most guidelines recommend similar adjuvant treatment approaches for all these stages despite the dearth of research focusing on high-risk stage III colon cancer and the potential for improved prognosis with intensive adjuvant treatment. Given the the proven efficacy of triplet chemotherapy in metastatic colorectal cancer treatment, the goal of this study is to evaluate the oncologic efficacy and safety of mFOLFIRINOX in comparison to those of the current standard of care, mFOLFOX 6, as an adjuvant treatment for patients diagnosed with high-risk stage III colon cancer after radical resection.

**Methods:**

This multicenter, randomized (1:1), open-label, phase II trial will assess and compare the effectiveness and toxicity of mFOLFIRINOX and mFOLFOX 6 in patients with high-risk stage III colon cancer after radical resection. The goal of the trial is to enroll 312 eligible patients, from 11 institutes, aged between 20 and 70 years, with an Eastern Cooperative Oncology Group (ECOG) performance status of 0–2, or between 70 and 75 with an ECOG performance status of 0. Patients will be randomized into two arms - Arm A, the experimental arm, and Arm B, the reference arm - and will receive 12 cycles of mFOLFIRINOX and mFOLFOX 6 every 2 weeks, respectively. The primary endpoint of this study is the 3-year disease-free survival, and secondary endpoints include the 3-year overall survival and treatment toxicity.

**Discussion:**

The Frost trial would help determine the oncologic efficacy and safety of adjuvant triplet chemotherapy for high-risk stage III colon cancers and ultimately improve prognoses.

**Trial registration:**

ClinicalTrials.gov NCT05179889, registered on 17 December 2021.

## Background

Based on numerous studies conducted since the publication of the MOSAIC trial in 2004 [1–3], the standard adjuvant treatment for stage III colon cancer (CC) following radical resection has evolved to involve doublet therapy consisting of oxaliplatin and 5-fluoropyromidine (5-FU). The addition of irinotecan to 5-FU for the treatment of radically resected stage III CC did not demonstrate improved survival compared to that achieved with 5-FU alone [4–6]; additionally, the combination of targeted agents, such as cetuximab or bevacizumab, with chemotherapy also failed to demonstrate remarkable oncological benefits [7–10]. Consequently, most international guidelines recommended a 6-month regimen of FOLFOX (oxaliplatin and 5-FU) or CAPOX (oxaliplatin and capecitabine) for adjuvant treatment of stage III CC.

Recently, the findings of the IDEA collaboration study [11] highlighted equivalent disease-free survival (DFS) rates with 3 months of CAPOX and with 6 months of CAPOX and substantially reduced toxicity with the former. Consequently, the National Comprehensive Cancer Network guidelines currently suggest 3 months of CAPOX or 3 or 6 months of FOLFOX as options for treatment of low-risk stage III (pT1-3N1) CC and 3–6 months of CAPOX or 6 months of FOLFOX for high-risk stage III (pT4N1-2 or TanyN2) CC treatment.

However, a paucity of information remains for patients with high-risk stage III CC, and research exploring more extensive adjuvant treatment options is scarce. According to the current guidelines, 3 months of CAPOX should be considered the first option for all high-risk stage II CC, low-risk stage III CC, and high-risk stage III CC. However, these patient groups exhibit considerably different prognoses. The IDEA study reported a 3-year DFS rate of 62.7–64% for patients with high-risk stage III CC compared to the 83.1–83.3% rate for patients with low-risk stage III CC [11]. Consequently, there is a need for research on more extensive adjuvant treatments for patients with high-risk stage III CC, even after successful R0 resection.

Personalized medicine is a crucial concept in the current treatment paradigm for metastatic colorectal cancer, and its extension to the management of non-metastatic colorectal cancer, particularly high-risk stage III CC, which carries high potential of progression to metastatic disease, is warranted. Therefore, it is essential to evaluate whether the triplet regimen of mFOLFIRINOX (oxaliplatin, irinotecan, and 5-FU), which has recently demonstrated superior efficacy in patients with unresectable metastatic colorectal cancer, can improve the prognosis of patients with high-risk stage 3 CC. Although a prospective single-arm study with a small sample size reported the safety and efficacy of triplet chemotherapy for locally advanced, resectable CC [12], and a randomized prospective study regarding a conventional regimen of triplet chemotherapy as an adjuvant treatment for high-risk stage III CC has been initiated in overseas [13], randomized controlled trials investigating the modified regimen of mFOLFIRINOX are lacking.

Therefore, the goal of this study is to evaluate the oncologic efficacy and safety of mFOLFIRINOX in comparison to those of the current standard of care, mFOLFOX 6, as an adjuvant treatment for patients diagnosed with high-risk stage III colon cancer after radical resection.

## Methods

### Study design

**Fig. 1:**
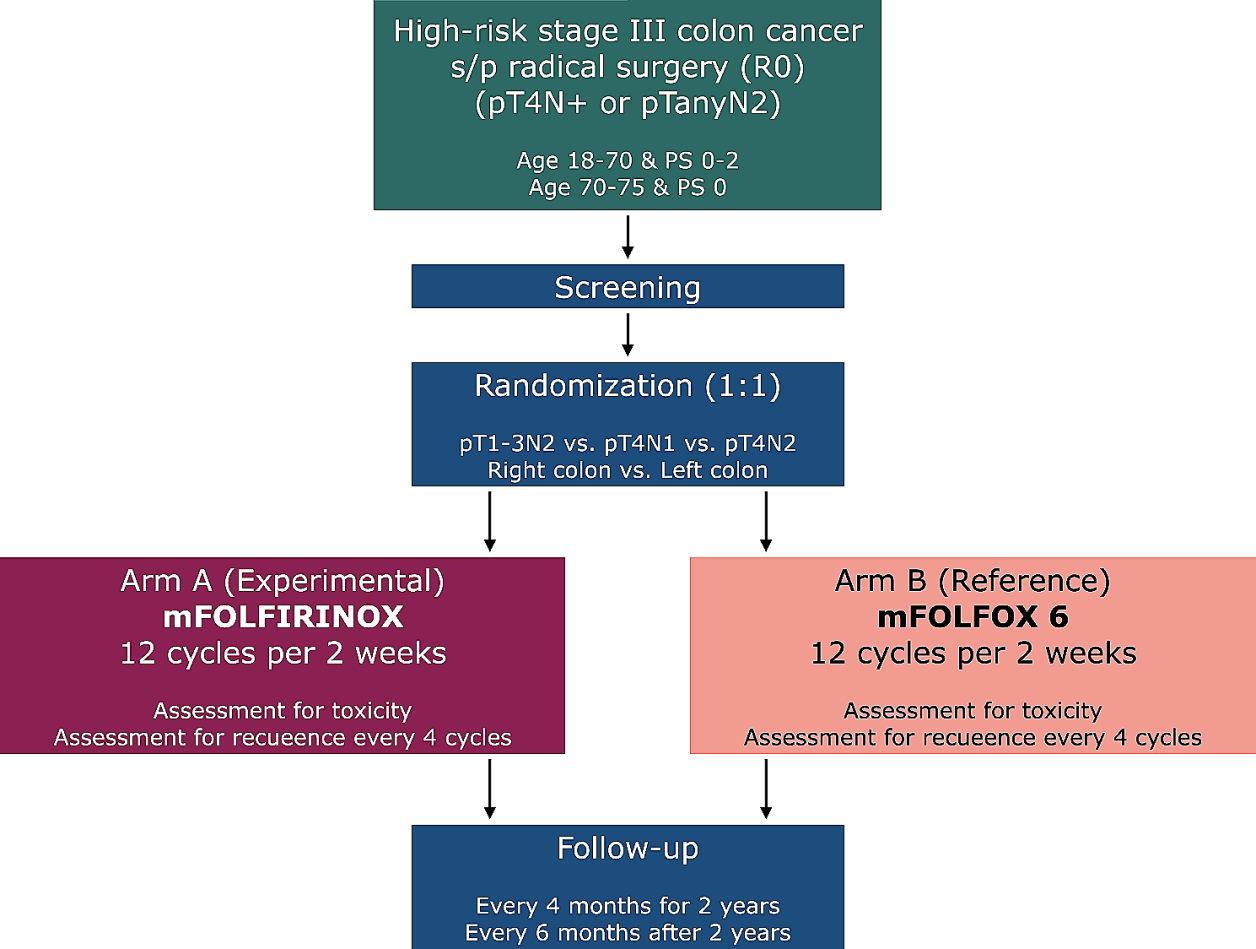
Flow-diagram of FROST trial, PS, performance score

This multicenter, randomized (1:1), open-label, phase II trial assesses the effectiveness and toxicity of mFOLFIRINOX compared to those of mFOLFOX-6 as an adjuvant treatment for patients with high-risk stage III CC. Patient enrollment will be performed across 11 tertiary hospitals in one country. The flow-diagram of the study is presented in Fig. 1.

### Study population

#### Inclusion criteria

- Age between 20 and 70 years with an Eastern Cooperative Oncology Group (ECOG) performance status of 2 or lower, or age between 71 and 75 years with an ECOG performance status of 0.
- Pathologically confirmed high-risk stage III CC (pT4N1-2 or pTanyN2).
- Successful R0 resection for CC above the upper rectum within 60 d prior to screening.
- Adequate organ functions, as determined by laboratory tests after operation and before screening.
  - absolute neutrophil count ≥ 2 × 10^6^ cells/mL.
  - hemoglobin ≥ 9.0 g/dL, platelet count ≥ 100 × 10^6^ cells/mL.
  - alanine aminotransferase/aspartate aminotransferase ≤ 2.5 times the upper limit of normal (ULN).
  - serum total bilirubin levels ≤ 1.5 times the ULN.
  - alkaline phosphatase levels ≤ 2.5 times the ULN.
  - serum creatinine levels ≤ 1.5 times the ULN or creatinine clearance > 50 mL/min based on the Cockcroft-Gault formula.
- Ability to understand and willingness to provide written informed consent.
- A life expectancy of at least 5 years.

#### Exclusion criteria

- Presence of distant metastases.
- Lower and middle rectal cancer requiring radiotherapy.
- Postoperative complications of grade 4 or higher according to the Clavien-Dindo classification.
- Underlying medical or systemic conditions that impede chemotherapy administration.
- Known hypersensitivity to all relevant tests or treatment components.
- Familial adenomatous polyposis or hereditary nonpolyposis colorectal cancer.
- Inflammatory bowel disease.
- Presence of other incurable prior malignancies.
- Pregnancy (confirmed by serum beta-human chorionic gonadotropin test) or lactation.
- Any other circumstances that, at the discretion of the investigator, might result in exclusion from the study.

### Interventions

#### Treatment and toxicity assessment

Patients will be randomized into two treatment arms - Arm A, the experimental arm, and arm B, the reference arm - and will receive 12 cycles of mFOLFIRINOX or mFOLFOX 6 every 2 weeks, respectively. The mFOLFIRINOX regimen consists of intravenous irinotecan 150 mg/m^2^, oxaliplatin 85 mg/m^2^, and leucovorin 400 (or levoleucovorin 200) mg/m^2^ on day 1 and continuous infusion of 5-FU 1200 mg/m^2^/d intravenously for the next 2 d (total 2400 mg/m^2^ over 48 h). The mFOLFOX 6 regimen consists of intravenous oxaliplatin 85 mg/m^2^, leucovorin 400 (or levoleucovorin 200) mg/m^2^, and bolus 5-FU 400 mg/m^2^ on day 1 and continuous infusion of 5-FU 1200 mg/m^2^/d intravenously for the next 2 d (total 2400 mg/m^2^ over 48 h).

Although there is no absolute contraindication with other treatments, caution will be exercised when co-administering these with drugs that might cause toxicities such as cardiotoxicity, hepatotoxicity, nephrotoxicity, myelodysfunction, and neurotoxicity. Toxicity will be monitored by taking the patient history and conducting laboratory tests before the initiation of the next cycle of treatment.

The drug dose could be adjusted at the discretion of the investigator, if necessary, because of toxicity, and the reduced dose could be sustained for subsequent cycles. Discontinuation will be considered after sustained toxicity despite dose reduction and management, recurrence during treatment, or on patient request.

#### Recurrence assessment

Recurrent assessment, including carcinoembryonic antigen measurement, abdominopelvic computer tomography (CT), and chest radiography or CT, will be performed after every 4 cycles of treatment and postoperatively every 4 months for 2 years and every 6 months for the next 3 years. The modality and interval of evaluation could be added and adjusted at the discretion of the investigator, if necessary. If recurrence is detected during treatment, study treatment will be discontinued and a standard personalized treatment will be conducted. If recurrence is detected during the follow-up period, a standard personalized treatment will be conducted. The participant timeline is presented in Table 1.

**Table 1:** Timeline of enrollment, intervention, assessment, and surveillance in FROST trial

Visit^1^	Screening	Chemotherapy period			Follow-up period	
		C1D1	C2–3D1 C5–7D1 C9–11D1	C4D1 C8D1 C12D1	F1–5	F6, F7
**Interval**			2 weeks (-4 d/+7 d)		4 months (-14/+30 d)	6 months (-14/+30 d)
Informed consent	X					
Screening number	X					
Complication check^2^	X					
Criteria check	X	X				
Randomization		X				
Performance status check	X	X	X	X		
Medical history and physical examination	X	X	X	X	X	X
Height, weight, BSA^3^		X	X	X		
Vital sign^4^	X	X	X	X		
Laboratory test^5^	X	X	X	X	X	X
Pregnancy test ^6^	X		X (even-numbered cycles)			
Treatment		X	X		X	
CEA^7^	X			X	X	X
Imaging study^8^				X	X	X
Colonoscopy^9^					X	X
**Safety assessment**		X	X	X		
**Efficacy assessment**				X	X	X

^1^The next visit is based on the previous visit date. In the treatment phase, the first day of each treatment cycle (e.g., C1D1, C2D1…) is the reference date

^2^Postoperative complications: Evaluate complications within 30 d after surgery according to the Clavien-Dindo Classification

^3^BSA (body surface area)

^4^Vital signs include blood pressure, heart rate, respiratory rate, and body temperature

^5^Laboratory tests include complete blood count, liver and kidney function tests, and electrolytes

^6^Pregnancy test (serum beta-human chorionic gonadotropin test): Performed on even-numbered cycles (C2, 4, 6, 8, 10, 12) only for fertile women

^7^CEA (carcinoembryonic antigen): The first test performed before surgery and during the postoperative screening period

^8^Imaging studies include abdominopelvic computed tomography (CT) and chest radiography (C4, C8, F2, F4, F6) or CT (C12, F1, F3, F5, F7)

^9^Colonoscopy is recommended in F1 and F7

### Endpoints

#### Primary endpoint

The primary endpoint of the study is the 3-year DFS rate based on intention-to-treat analysis, which is defined as the proportion of patients who were alive without recurrence at 3 years from the date of surgery. It will be calculated using the time from the date of surgery to the date of recurrence detection, death, or the last follow-up. In cases where patients are lost before the 3-year mark, their data will be censored as of the last follow-up date and assessed accordingly.

#### Secondary endpoints

The secondary endpoints are the 3-year overall survival (OS) rate based on intent-to treat analysis, which is defined as the proportion of patients who are still alive 3 years from the date of surgery and calculated using the time from the date of surgery to the date of death or last follow-up; treatment-related toxicity according to the CTCAE (Common Terminology Criteria for Adverse Events) version 5.0; treatment discontinuation rate, defined as the proportion of participants in each arm who discontinued treatment because of toxicity or lack of compliance; and the percentage of the drug dose actually administered relative to the target drug dose. These secondary endpoints will provide valuable insights into the safety and tolerability of the adjuvant treatments compared in this study.

### Randomization

Randomization will be adjusted based on the following stratification factors: pT1-3N2 versus pT4N1 versus pT4N2, and the right colon (from the cecum to the distal transverse colon above the splenic flexure colon) versus the left colon (from the splenic flexure colon to the rectosigmoid colon). This process will be conducted according to the special program on the electronic case report form (eCRF) webpage, and the result of randomization will be presented on the webpage immediately after the registration of patients. This eCRF, which includes the randomization program, was developed by the special department, the “Academic Clinical Research Operating Support System (ACROSS)” of the lead institute, which consists of data management and monitoring specialists that support investigator-oriented clinical studies. They are independent from the sponsor, and no competing interests will exist.

### Data management

To conceal personal information, the allocation sequence employs the double-digit participant number and the three-digit institute number, which are automatically generated on the eCRF in order of registered participants, based on the date of acquisition of informed consent. Access to the eCRF will be exclusively granted to registered researchers involved in this study. Screening, obtaining informed consent, enrollment, registration, and data collection will be conducted by investigators or qualified clinical research coordinators. Blinding will not be implemented because of the open-label nature of the study.

### Monitoring

#### Data safety monitoring

Completeness of data collection will be assessed when 20% of the sample completes treatment, and improvements will be made, if necessary. Treatment completion rates and rates of the incidence of serious adverse events (SAE) will be evaluated at the end of treatment for 20% and 40% of the sample, respectively.

#### Safety monitoring

In the event of an SAE occurring at any institution, the contents shall be promptly reported to the Institutional Review Board (IRB) of the leading and corresponding institutions using the prescribed form. If no SAEs occur, monitoring results will be included in the final report. Any protocol violations, non-compliance, or unexpected problems will also be reported to the IRB of the leading and corresponding institutes. If more than five SAE are reported at any institution, the principal investigator, along with the co-investigators, will discuss the study’s sustainability and the need to potentially discontinue or modify the study. The results of this discussion will be reported to the IRB. In the event of participants experiencing physical damage as a result of participating in this study, the principal investigator shall provide compensation in accordance with the provisions of relevant laws and regulations.

#### Auditing

The ACROSS team will monitor data collection and protocol adherence. Auditing of investigators will be conducted at least annually and may be conducted at additional times, if necessary.

### Statistical analysis

#### Sample size calculation

Based on previous research [11], it is anticipated that the experimental group receiving mFOLFIRINOX as adjuvant treatment will achieve a 3-year DFS rate of 77.5%, with an improvement of 12.5% compared to the control group, which is estimated to have a 65% DFS rate. To determine the sample size for the study, a power analysis was performed using the PASS (Power Analysis and Sample Size) 13.0 program (NCSS, LLC, Kaysville, Utah, USA). The calculations indicated that a minimum of 156 subjects will be required for each treatment arm, considering the desired statistical power of 80%, the significance level of 10%, and the dropout rate of 10%. Therefore, a total of 312 subjects, equally stratified and randomized, will be enrolled to ensure adequate statistical power and robust results.

#### Outcome assumptions

Statistical analyses for primary and secondary outcomes of the intent-to-treat group and per-protocol group will be performed using SPSS version 26 (IBM, Armonk, NY, USA).

### Analysis of prognostic factors and biomarkers

Prognostic factors and biomarkers will be analyzed in this study. Information about various clinical, pathological, and molecular characteristics, including microsatellite instability, KRAS, NRAS, BRAF, and HER2 status, of the enrolled patients will be collected and assessed. The analysis of these factors will provide valuable insights into the prognosis and potential treatment response of patients with high-risk stage III CC. Through these analyses, we will identify molecular markers that might affect disease progression and treatment outcomes. This comprehensive analysis of prognostic factors and biomarkers will enhance our understanding of the underlying biology of high-risk stage III CC and could reveal important associations between these molecular characteristics and clinical outcomes, thereby guiding personalized treatment and optimized patient management.

## Discussion

Despite their poor outcomes, there is a considerable lack of research regarding the prognosis and treatment outcomes of patients diagnosed with high-risk stage III CC. Although triplet chemotherapy holds theoretical promise for providing more intensive systemic control in this patient population, its safety and oncological superiority need to be established through prospective randomized controlled studies.

The conventional triplet chemotherapy regimen (oxaliplatin 85 mg/m^2^, irinotecan 180 mg/m^2^, and 5-FU 2400 mg/m^2^) is associated with considerably higher toxicity than is the modified regimen (oxaliplatin 85 mg/m^2^, irinotecan 150 mg/m^2^, and 5-FU 2400 mg/m^2^). Therefore, the conventional regimen may not be suitable for Asian patients, as it may potentially result in poor oncological outcomes because of decreased rate of completion of the planned treatment schedule, reduced drug dose, and poor compliance [14].

The goal of this study is to assess the effectiveness and toxicity of mFOLFIRINOX and mFOLFOX 6 in 312 patients who have been diagnosed with high-risk stage III CC after radical resection.

We expect that our findings will help elucidate the oncologic efficacy and safety of adjuvant triplet chemotherapy for patients with high-risk stage III CC and provide useful information regarding this treatment. Ultimately, the findings of this study will improve the prognosis and refine personalized treatment approaches for patients with high-risk stage III CC.

## Trial status

The first patient was enrolled in June 2021, and as of July 2023, 19 patients have been enrolled from a single institute. Based on the expectation that 9 additional sites will initiate enrollment within 3 months, patient enrollment is expected to be completed by 2026. The last updated protocol was version 4.0. Protocol amendments will be conducted after sufficient discussion and agreement of all investigators, only if absolutely necessary.

## World health organization trial registration data set

**Table:** 

Data category	Information
Primary registry and trial identifying number	ClinicalTrials.gov NCT05179889
Date of registration in primary registry	17 December 2021
Secondary identifying numbers	2020-11-062
Source(s) of monetary or material support	Boryung Pharmaceutical Co., Ltd.
Primary sponsor	Korean Society of Coloproctology
Secondary sponsor(s)	Korea National Enterprise for Clinical Trial
Contact for public queries	Ji Yeon Kim, M.D. Phone: +82-42-280-7175 FAX: +82-42-257-8024 Email: jkim@cnu.ac.kr
Contact for scientific queries	Ji Yeon Kim, M.D. Department of Colorectal Surgery, Chungnam National University Hospital & College of Medicine, Daejeon, Korea
Public title	mFOLFIRINOX versus mFOLFOX 6 as adjuvant treatment for high-risk stage III colon cancer - the FROST study: Study protocol for a multicenter, randomized controlled, phase II trial
Scientific title	mFOLFIRINOX versus mFOLFOX 6 as adjuvant treatment for high-risk stage III colon cancer - the FROST study: Study protocol for a multicenter, randomized controlled, phase II trial
Countries of recruitment	South Korea
Health condition(s) or problem(s) studied	Colon cancers
Intervention(s)	Arm A, the experimental arm: 12 cycles of mFOLFIRINOX Arm B, the reference arm: 12 cycles of mFOLFOX 6
Key inclusion and exclusion criteria	Inclusion criteria -Age between 20 and 70 years with an Eastern Cooperative Oncology Group (ECOG) performance status of 2 or lower, or age between 71 and 75 years with an ECOG performance status of 0 -Pathologically confirmed high-risk stage III CC (pT4N1-2 or pTanyN2) -Successful R0 resection for CC above the upper rectum within 60 d prior to screening -Adequate organ functions, as determined by laboratory tests after operation and before screening (Absolute neutrophil count ≥ 2 × 106 cells/mL, hemoglobin ≥ 9.0 g/dL, platelet count ≥ 100 × 106 cells/mL, alanine aminotransferase/aspartate aminotransferase ≤ 2.5 times the upper limit of normal (ULN), serum total bilirubin levels ≤ 1.5 times the ULN, alkaline phosphatase levels ≤ 2.5 times the ULN, serum creatinine levels ≤ 1.5 times the ULN or creatinine clearance > 50 mL/min based on the Cockcroft-Gault formula -Ability to understand and willingness to provide written informed consent -A life expectancy of at least 5 years Exclusion criteria -Presence of distant metastases -Lower and middle rectal cancer requiring radiotherapy -Postoperative complications of grade 4 or higher according to the Clavien-Dindo classification -Underlying medical or systemic conditions that impede chemotherapy administration -Known hypersensitivity to all relevant tests or treatment components -Familial adenomatous polyposis or hereditary nonpolyposis colorectal cancer -Inflammatory bowel disease -Presence of other incurable prior malignancies -Pregnancy (confirmed by serum beta-human chorionic gonadotropin test) or lactation Any other circumstances that, at the discretion of the investigator, might result in exclusion from the study
Study type	Multicenter, randomized (1:1), open-label, phase II trial
Date of first enrolment	June 2021
Target sample size	312
Recruitment status	Recruiting
Primary outcome(s)	3-year DFS rate based on intention-to-treat analysis
Key secondary outcomes	3-year overall survival (OS) rate, treatment-related toxicity

## Data Availability

The datasets used and/or analyzed during the current study will be available from the corresponding author upon reasonable request.

## Abbreviations

CC: Colon cancer
5-FU: 5-Fluoropyromidine
CAPOX: Capecitabine and oxaliplatin
DFS: Disease-free survival
ULN: Upper limit of normal
IRB: Institutional review board
SAE: Serious adverse events
